# POCU1b, the n-Butanol Soluble Fraction of Polygoni Cuspidati Rhizoma et Radix, Attenuates Obesity, Non-Alcoholic Fatty Liver, and Insulin Resistance via Inhibitions of Pancreatic Lipase, cAMP-Dependent PDE Activity, AMPK Activation, and SOCS-3 Suppression

**DOI:** 10.3390/nu12123612

**Published:** 2020-11-24

**Authors:** Junghyun Kim, Chan-Sik Kim, Kyuhyung Jo, Ik Soo Lee, Joo-Hwan Kim, Jin Sook Kim

**Affiliations:** 1 Herbal Medicine Research Division, Korea Institute of Oriental Medicine, Daejeon 34054, Korea; dvmhyun@jnbu.ac.kr (J.K.); jopd7414@kiom.re.kr (K.J.); knifer48@kiom.re.kr (I.S.L.); 2 Department of Oral Pathology, School of Dentistry, Jeonbuk National University, Jeonju 54896, Korea; 3 Clinical Medicine Division, Korea Institute of Oriental Medicine, Daejeon 34054, Korea; chskim@kiom.re.kr; 4 Department of Life Science, Gachon University, 1342, Seongnamdaero, Gyeonggido 13120, Korea; kimjh2009@gachon.ac.kr

**Keywords:** obesity, non-alcoholic fatty liver, insulin resistance, *n*-BuOH soluble fraction of Polygoni Cuspidati (POCU1b), cyclic adenosine monophosphate, phosphodiesterase, AMPK, SOCS-3, high-fat fed rats

## Abstract

This study investigated the effects of the *n*-BuOH soluble fraction of Polygoni Cuspidati 80% ethanol extract (POCU1b) on high-fat diet (HFD)-induced obesity, non-alcoholic fatty liver (NAFL), and insulin resistance (IR) to find a safe and more effective agent. HPLC profiling of POCU1b identified seven marker compounds. POCU1b increased glycerol release, cyclic adenosine monophosphate (cAMP) level, and inhibited phosphodiesterase (PDE) activity. Seven weeks of POCU1b treatment decreased body weight gain, weight and adipocyte size in fat tissues, serum lipids, and triglyceride and lipid droplets in the livers of HFD-fed rats. POCU1b improved blood glucose, insulin sensitivity, and impaired insulin secretion in the pancreas. Further, POCU1b ameliorated adiponectin, leptin, IL-6 and TNF-α levels, increased AMPK and p-ACC expression, activated CPT-1 activity, and suppressed *FAS* mRNA, SOCS-3 protein expression, and NF-κB DNA-binding activity. When compared with the Xenical^®^-treated group, a positive group, the action of POCU1b on body weight was more effective than that of Xenical. POCU1b did not show side effects, such as oily spotting and loss of appetite. These results suggest that POCU1b possesses therapeutic or preventive potential for obesity, NAFL and IR via inhibitions of pancreatic lipase and cAMP-dependent PDE activity, AMPK activation, and SOCS-3 suppression, without oily spotting and loss of appetite.

## 1. Introduction

Obesity is a growing public health concern worldwide, and excess fat deposition in the liver is accompanied by histological alterations, varying from simple hepatic steatosis and non-alcoholic fatty liver (NAFL) to non-alcoholic steatohepatitis (NASH), that can progress to cirrhosis and liver cancer, and is also a risk factor of insulin resistance (IR) [1]. The prevalence of non-alcoholic fatty liver disease (NAFLD) among 4437 Korean patients with type 2 diabetes was reported to be 72.7% [2]. Even individuals with IR but no diabetes have higher levels of liver fat. Obesity, NAFLD, and IR commonly exist together [3]. The consumption of foods with high energy and dietary fat promotes body fat storage in humans and animals. The inhibition of the digestion and absorption of dietary fat has been used as a strategy to treat obesity. Pancreatic lipase is the most important enzyme for the digestion of dietary triglycerides (TGs) [4]. Lipolysis is an important mechanism to reduce body fat, and involves the lipase-catalyzed hydrolysis of TG to glycerol and free fatty acids. The lipolytic effects of phosphodiesterase (PDE) inhibitors are mediated by cyclic adenosine monophosphate (cAMP)-dependent PDE inhibiting, and the subsequent increase in cAMP levels stimulates hormone-sensitive lipase (HSL) to reduce the lipogenesis [5]. To control obesity and minimize complications, lifestyle modification and the acquisition of healthy food habits and medicine are needed. Sibutramine, rimonabant, and orlistat (Xenical^®^) are approved by the US FDA for the treatment of obesity. However, sibutramine and rimonabant are no longer marketed because of serious side effects, such as cardiovascular problems, insomnia, psychiatric action, and suicidal ideation [6]. Orlistat, a potent natural inhibitor of pancreatic lipases, promotes body weight loss and reduces the incidence of diabetes by nearly 40% in obese people [7]. However, orlistat also has serious side effects, such as steatorrhea, stomach pain, irregular menstrual periods, bowel urgency, and headaches [8,9]. Thus, there clearly is an urgent need for safer and more effective anti-obesity agents for long-term treatment.

Various herbal extracts and phytochemicals have been shown to inhibit pancreatic lipase or to stimulate lipolysis via the inhibition of PDE [10,11]. Dried rhizoma et radix of Polygonum cuspidatum Siebold et Zuccarinii (Polygonaceae) has been widely used in Korea, China, Japan, and Taiwan for the treatment of constipation, gallstones, hepatitis, and inflammation [12]. It has been confirmed to have antibacterial activity against *Streptococcus mutans* for oral hygiene [13,14,15] and anti-diabetic action [16]. We previously reported that the *n*-BuOH soluble fraction of Polygoni Cuspidati 80% EtOH extract (POCU1b) inhibits pancreatic lipase activity and adipocyte differentiation in 3T3-L1 preadipocytes by reducing lipid droplet formation and glycerol-3-phosphate dehydrogenase activity, and increasing p-AMPK level [17]. The mRNA and protein expression of adipocyte differentiation-related protein (ADRP), perilipin, peroxisome proliferator-activated receptor-gamma (PPAR-γ), and CCAAT/enhancer-binding protein-α were also reduced after POCU1b treatment in 3T3-L1 cells [17]. *P. cuspidatum* root 50% ethanol extract has been reported to exert an anti-obesity effect via downregulation of sterol regulatory element-binding protein-1c, PPAR-γ, adipocyte protein 2, fatty acid synthase (FAS), and acetyl CoA carboxylase (ACC), as well as anti-oxidant action in 3T3-L1 adipocytes [18].

Based on the inhibitory action of POCU1b on pancreatic lipase in vitro [17], the present study investigated whether POCU1b inhibits fat absorption in vivo, affects an adrenoceptor-mediated glycerol output using rat adipocytes, and promotes lipolysis via an inhibitory effect on cAMP-dependent PDE activities ex vivo and in vitro. Furthermore, we investigated whether seven weeks of POCU1b treatment attenuates obesity, NAFL, and IR using high-fat diet (HFD)-fed rats, by measuring the body weight, fat weight, adipocyte size, liver TG levels, and insulin sensitivity, and determining the mRNA or protein expression of (p)-AMPK and suppressor of cytokine signaling-3 (SOCS-3) in the liver.

## 2. Materials and Methods

### 2.1. Preparation and High Performance Liquid Chromatography (HPLC) Analysis of POCU1b

Polygoni Cuspidati rhizoma et radix was purchased from a traditional herbal medicine store, Baekjaedang (Daejeon, Korea), in 2009, and identified by Professor J.-H. Kim at the Department of Life Science, Gachon University. The voucher specimen (KIOM-POCU-2009) was deposited at the Korea Institute of Oriental Medicine. As reported [17], Polygoni Cuspidati rhizoma et radix was extracted with 80% EtOH at room temperature for seven days, dried, suspended in H_2_O, and then partitioned successively with equal volumes of *n*-hexane, EtOAc, and *n*-BuOH to yield *n*-hexane, EtOAc, and *n*-BuOH soluble fractions, respectively. The *n*-BuOH soluble fraction (POCU1b) was subjected to a series of chromatographic techniques, leading to the isolation of seven marker compounds. The chemical content of POCU1b was analyzed by HPLC using an Agilent 1200 HPLC (Agilent Technologies, Palo Alto, CA, USA) equipped with a binary pump, vacuum degasser, autosampler, column compartment, and diode array detector. The column used was a Spherex C18 (5.0 µm, 4.6 × 250 mm, Phenomenex, Torrance, CA, USA). The mobile phase was a mixture of acetonitrile (Fisher Chemicals, Fair Lawn, NJ, USA) (solvent A) and water (J.T. Baker, Phillipsburg, NJ, USA) with 0.1% acetic acid (Wako, Tokyo, Japan) (solvent B). A linear gradient elution was performed from 97% to 70% B in 40 min, from 70% to 30% B in 20 min, and from 30% to 0% B in 5 min, followed by washing and reconditioning of the column. The column temperature was maintained at 30 °C. The flow rate was 1.0 mL/min, the detection wavelength was at 290 nm, and the injection volume was 10 μL. POCU1b (5 mg) was dissolved in MeOH (10 mL), and the solution was filtered through a 0.2 μm syringe filter (Millipore, Bedford, MA, USA) prior to injection. Seven standard stock solutions of 1 mg/mL were prepared in MeOH and filtered through a syringe filter prior to injection. Resveratrol, resveratrol-3-*O*-β-d-glucopyranoside, emodin-8-*O*-β-d-glucopyranoside, and emodin were purchased from Sigma-Aldrich (St. Louis, MO, USA). Emodin-1-*O*-β-d-glucopyranoside, torachrysone-8-*O*-β-d-glucopyranoside, and physcion-8-*O*-β-d-glucopyranoside were purchased from Chengdu Must BioTechnology Co. Ltd. (Chengdu, China).

### 2.2. Short-Term Animal Study for Serum Triglyceride (TG) Levels After Oral Administration of a Lipid Emulsion

Wistar male rats (Orient Bio Inc., Gyeonggi-do, Korea) were housed under a 12 h light/dark cycle in a temperature- and humidity-controlled room for one week with free access to food and water. After acclimation for one week, the rats (seven weeks old and a body weight of 250 g) were divided into six groups. The rats were fasted overnight and were then orally administrated 3 mL of a lipid emulsion consisting of corn oil (6 mL), cholic acid (80 mg), cholesteryloleate (2 g), and saline solution (6 mL) with or without POCU1b (10, 25, or 50 mg/kg). Xenical^®^ (Roche pharma Ltd., Reinach, Swiss, 45 mg/kg) was used as a positive control. Blood was collected from the tail vein 0, 1, 2, 3, and 4 h after oral administration and centrifuged at 5500× *g* for 5 min. The TG levels were determined using a TG test kit (Wako, Osaka, Japan). All animal procedures were approved by the Institutional Animal Care and Use Committee of the KIOM (approval number: 09-186).

### 2.3. Preparation of Fat Pads and Measurements of Glycerol Release and Cyclic Adenosine Monophosphate (cAMP) 

The fat pads from Wistar male rats (Orient Bio Inc., Gyeonggi-do, Korea) were prepared as follows: after acclimation for one week, seven-week-old rats were killed by cervical dislocation and their epididymal adipose tissue was quickly removed. The tissues were rinsed with 0.9% NaCl and placed in 9 mL of Krebs-Ringer phosphate buffer (pH 7.4) containing 25 mg of purified collagenase. The mixture was incubated at 37 °C for 90 min. After incubation, the cells were freed by gentle agitation, collected by centrifugation at 500× *g*, and washed four times with albumin-free Krebs-Ringer phosphate buffer. Finally, the cells were diluted to a convenient volume with buffer.

For the glycerol release assay, adipocytes were treated with 200 μL of Hanks balanced solution (HBS) supplemented with 2.5% bovine serum albumin (Sigma). The adipocytes were incubated with 0.8 mL of glycerol reagent (Sigma) and 10 μg/mL of isoproterenol (TCI, Tokyo, Japan), 1 μg/mL of propranolol (Sigma), or POCU1b (0.1, 1, or 10 μg/mL) at 37 °C for 1 h. The released glycerol was assayed using free glycerol reagent. The absorbance of the solution at 540 nm was measured using a microplate reader (BioTek, Synergy HT, Winooski, VT, USA), and glycerol release was calculated according to the following formula:

glycerol = (abs. of sample – abs. of blank) / (abs. of standard – abs. of blank) × concentration of standard

For the cAMP assay, the intracellular cAMP level was measured using a cAMP assay kit (Sigma) according to the manufacturer’s instructions. Briefly, adipocytes incubated with the adrenoceptor agonist, isoproterenol, or POCU1b for 1 h were washed with phosphate-buffered saline and dissolved in lysis reagent. The samples were transferred into a plate coated with a cAMP-peroxidase conjugate, followed by addition of the enzyme substrate, and the colorimetrical density was measured. All animal procedures were approved by the Institutional Animal Care and Use Committee of the KIOM (approval number: 09-186).

### 2.4. Phosphodiesterase (PDE) Activity Assay

PDE activity was assayed using the PDE-GloTM phosphodiesterase activity kit (Promega Corp., Fitchburg, WI, USA) according to the manufacturer’s instructions. Briefly, PDE enzyme, POCU1b or non-specific PDE inhibitor, and 3-isobutyl-1-methoxyxanthin (IBMX, Sigma, St. Louis, MO, USA) were incubated with 1 μM of cAMP to initiate the PDE reaction. The PDE-GloTM termination buffer containing IMBX and the detection solution was added and mixed well. The Kinase-Glo reagent was added and incubated at room temperature for 10 min. Luminescence at 405 nm was measured using the BioTek plate-reader.

### 2.5. Long-Term Animal Study of Lipolytic Effect

After acclimation for one week, SD male rats (Orient Bio Inc., Gyeonggi-do, Korea, four weeks old) were fed a high-fat diet (HFD) containing 45% fat (D12451; Research Diet, Inc., New Brunswick, NJ, USA) for 13 weeks. Then, HFD-fed rats and age-matched normal rats were randomly divided into five groups, and received one of the following diets for seven weeks without exercise: normal diet (NOR group), high-fat diet (HFD group), HFD containing 0.1% Xenical^®^ (XEN group, positive control), and HFD containing 0.1% or 1.0% POCU1b (POCU1b 0.1, POCU1b 1.0 groups). Each group included 10 rats. Body weight and the total amount of food consumed by each rat were measured once a week. After seven weeks, the rats were killed under deep anesthesia and blood was collected by heart puncture. Serum was prepared and stored at −80 °C until analysis. Adipose tissues and the liver were quickly removed and weighed, and one part was flash-frozen in liquid nitrogen and stored at −80 °C until analysis. One part was fixed in neutralized formalin for histological analysis. All animal procedures were approved by the Institutional Animal Care and Use Committee of Orient Bio Inc. (approval number: SN08023).

### 2.6. Serum Analysis

Total cholesterol (TC), triglycerides (TG), high-density lipoprotein (HDL) cholesterol, low-density lipoprotein (LDL) cholesterol, free fatty acids, and glucose in the blood were measured using an automated analyzer (Hitachi, Ltd., Tokyo, Japan).

### 2.7. Liver TG Measurement

Liver tissue (0.5 g) was homogenized in 4.5 mL of Krebs Ringer phosphate buffer. The homogenate (0.2 mL) was extracted with 4 mL of chloroform/methanol (2:1, *v/v*). The extract was concentrated under a nitrogen stream. The residue was analyzed using a commercial kit (Asan Pharm. Co., Seoul, Korea).

### 2.8. Enzyme-Linked Immunosorbent Assay (ELISA)

Serum adiponectin, leptin, insulin, tumor necrosis factor (TNF)-α, and interleukin (IL)-6 levels were quantified using commercial ELISA kits (MyBioSource, San Diego, CA, USA).

### 2.9. Carnitine Palmitoyl Transferase (CPT)-1 Activity Assay

To isolate mitochondria, fresh liver tissue was minced on ice and homogenized in buffer 1 (220 mM of mannitol, 70 mM of sucrose, 20 mM of Tris-HCl, and 1 mM of ethylene diamine tetra acetic acid (EDTA)) 1:6 (w/v). The homogenate was centrifuged at 22,500× *g* at 4 °C for 25 min. After removing the fat layer, the pellet was resuspended and the suspension was centrifuged at 700× *g* at 4 °C for 10 min. The pellet was discarded and the supernatant was re-centrifuged at 15,000× *g* at 4 °C for 30 min to pellet the mitochondria, which were immediately used for the CPT-1 assay. CPT-1 activity was assayed spectrophotometrically following carnitine-dependent CoA liberation in the presence of palmitoyl-CoA and 5,5′-dithiobis-(2-nitrobenzoic acid) (DTNB) at 412 nM. The reaction buffer was: 116 mM of Tris-HCl, 0.09% Triton, 1.1 mM of EDTA, 35 µM of palmitoyl-CoA, 0.12 mM of DTNB, and 3.3 mM of carnitine in a final volume of 0.9 mL. A buffer without mitochondria was used as a blank. Mitochondrial proteins (100 µg) were brought to a final concentration of 1 µg/µL in buffer 1 containing 0.1% Triton, added to the assay buffer, and incubated for 30 s. Then, the reaction was started. Changes in absorbance were monitored for 3 min, and CPT-1 activity was expressed as nM CoA/min × mg protein.

### 2.10. Western Blot Analysis

Liver tissues (0.1–0.2 g) were lysed in a solution containing 250 mM of sucrose, 1 mM of EDTA, 0.1 mM of phenylmethylsulfonyl fluoride, and 20 mM of potassium phosphate buffer at pH 7.6, using a homogenizer at 3000 rpm. Equal amounts of proteins (50 μg) were subjected to immunoblotting with antibodies against TNF-α, IL-6, adenosine monophosphate-activated protein kinase (AMPK), phosphorylated (p)-AMPK, p-acetyl-CoA carboxylase (p-ACC), suppressor of cytokine signaling-3 (SOSC-3, Santa Cruz Biotechnology, Santa Cruz, CA, USA), and β-actin (Sigma). Bound horseradish peroxidase-conjugated secondary antibody was detected using an enhanced chemiluminescence detection system (iNtRON Biotechnology, Daejeon, Korea). Protein levels were determined by analyzing the signals captured on the polyvinylidene difluoride membranes using an image analyzer (Las-3000, Fuji Photo, Tokyo, Japan).

### 2.11. RNA Isolation and Reverse Transcription (RT)-PCR

Total RNA was isolated using TRIzol reagent (MCRC, Cincinnati, OH, USA) according to the manufacturer’s instructions; cDNA was synthesized from 3 μg of RNA using RT-premix (Bioneer, Daejeon, Korea) in a TaKaRa PCR Thermal Cycler (TaKaRa Bio, Shiga, Japan). RT-PCR products were separated by electrophoresis. DNA band intensities in 1.2% agarose gels containing ethidium bromide were quantitated by densitometry (Las-3000).

### 2.12. Histological Analysis

The dissected tissues were fixed in 10% neutral buffered formalin for histological analysis and embedded in paraffin. Paraffin-embedded sections were cut at a thickness of 4 μm and stained with hematoxylin and eosin (H&E). Adipocyte sizes were measured in random microscopic areas from independent animals using an Olympus BX51 microscope system (Olympus, Tokyo, Japan).

### 2.13. Immunohistochemistry

Paraffin sections were deparaffinized, hydrated, and treated with 1% H_2_O_2_ in methanol. The sections were incubated with anti-insulin antibody (Santa Cruz Biotechnology Inc., Dallas, TX, USA) at room temperature for 2 h using a standard manual immunoperoxidase procedure with streptavidin-peroxidase (LSAB kit, Dako, Carpinteria, CA, USA). The stained sections were observed under a light microscope equipped with an Olympus DP 71 camera (Olympus corporation, Tokyo, Japan). The staining intensity was analyzed using Image J software (NIH, Bethesda, MD, USA).

### 2.14. Measurment of NF-κB DNA Binding Using an ELISA-Based Method

Nuclear proteins were isolated using commercially available nuclear and cytoplasmic extraction kits (Pierce, Rockford, IL, USA). NF-κB DNA-binding activity was evaluated using an ELISA-based EZ-Detect^TM^ Transcription Factor Kit for NF-κB p65 (Thermo Fisher Scientific InC., Rockford, IL, USA) according to the manufacturer’s instructions. The amount of active NF-κB p65 was obtained from a standard curve and normalized to the protein content. 

### 2.15. Statistical Analysis

Image analysis was conducted using Image J software (NIH, Bethesda, Maryland, USA) and values were averaged. Data are expressed as the mean ± standard error (SEM). Means were compared using Student’s t-test or one-way ANOVA, followed by Dunnett’s multiple comparison test on the Prism 7.0 software (GraphPad software, San Diego, CA, USA); *p* < 0.05 was considered significant.

## 3. Results

### 3.1. Preparation and HPLC Analysis of POCU1b

**Figure 1:**
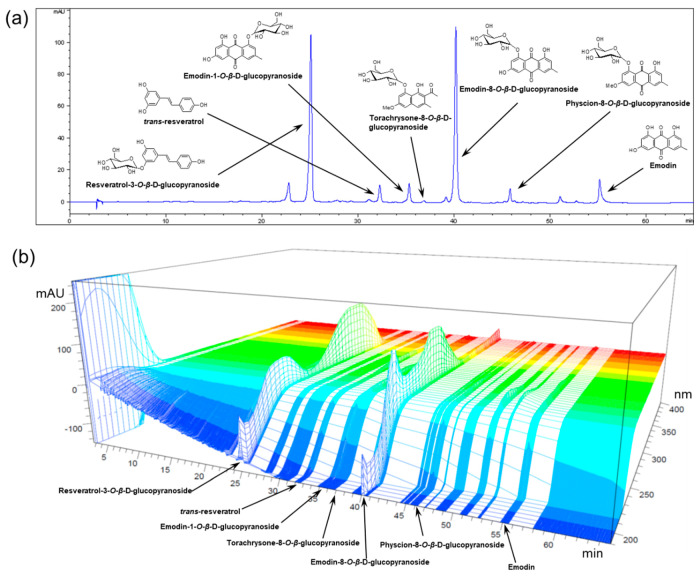
HPLC chromatograms of POCU1b. (**a**) One-dimensional (290 nm) and (**b**) three-dimensional (200–400 nm) HPLC chromatograms of POCU1b.

Dried Polygoni Cuspidati rhizoma et radix (5.5 kg) was extracted with 80% EtOH to afford POCU (590 g), which was partitioned successively with *n*-hexane, EtOAc, and *n*-BuOH to yield *n*-hexane (35 g), EtOAc (212 g), and *n*-BuOH (105 g) soluble fractions, respectively. Among these fractions, the *n*-BuOH soluble fraction (POCU1b) was selected for further study because of its strongest inhibitory effect on pancreatic lipase activity [17]. Seven marker compounds of POCU1b were identified as resveratrol, resveratrol-3-*O*-β-d-glucopyranoside, emodin-1-*O*-β-d-glucopyranoside, torachrysone-8-*O*-β-d-glucopyranoside, emodin-8-*O*-β-d-glucopyranoside, physcion-8-*O*-β-d-glucopyranoside, and emodin by comparing their physicochemical and spectral data with those in the literature [19,20]. For the standardization of POCU1b, quantitative data of the marker compounds was obtained by comparing the retention times and areas with those of standard compounds (Figure 1, Table 1).

### 3.2. Effects of POCU1b on Levels of Serum TG and cAMP, Glycerol Release, and PDE Activity

**Figure 2:**
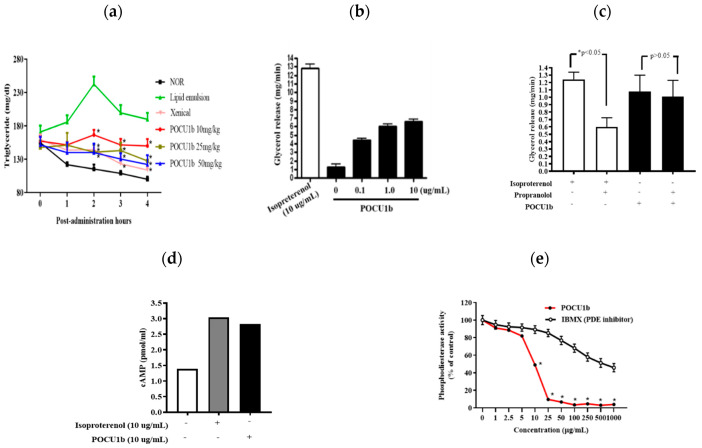
Effects of POCU1b on levels of serum triglyceride (TG), glycerol release, cyclic adenosine monophosphate (cAMP), and phosphodiesterase (PDE) activity. (**a**) After acclimation, seven-week-old rats were fasted and administrated with a lipid emulsion with or without POCU1b. Xenical^®^ (Roche pharma Ltd., Reinach, Swiss, 45 mg/kg) was used as a reference. Blood was collected from the tail vein. POCU1b significantly reduced the serum TG levels at 2, 3, and 4 h after oral administration. * *p* < 0.05 vs. lipid emulsion. (**b**,**c**) Rat epididymal adipocytes were incubated with a glycerol reagent and isoproterenol (adrenoceptor agonist), propranolol (adrenoceptor antagonist), or POCU1b at 37 °C for 1 h. The absorbance of the solution at 540 nm was measured using a microplate reader. (**b**) Glycerol release was dose-dependently increased by POCU1b treatment. (**c**) POCU1b-induced glycerol release was not decreased by the co-treatment with propanolol. * *p* < 0.05 vs. isoproterenol. (**d**) Rat epididymal adipocytes were incubated with (+) or without (−) isoproterenol or POCU1b for 1 h, transferred into a plate coated with a cAMP-peroxidase conjugate, the enzyme substrate was added, and the colorimetrical density was measured. POCU1b and isoproterenol increased cAMP levels. (**e**) PDE enzyme and POCU1b or IBMX (PDE inhibitor) were incubated with 1 μM of cAMP. After addition of the termination buffer, the Kinase-Glo reagent was added and incubated at room temperature for 10 min. Luminescence at 405 nm was measured. IC_50_ values for POCU1b and IBMX were 10.1 μg/mL and 667.0 μg/mL, respectively. * *p* < 0.05 vs. IBMX.

As previously published, POCU1b directly inhibited pancreatic lipase activity in vitro [17]. In the present study, we investigated whether POCU1b prevents the absorption of lipid from the small intestine after oral administration of a lipid emulsion to rats. POCU1b dose-dependently reduced the serum TG levels at 2, 3, and 4 h after treatment with a lipid emulsion (Figure 2a). Next, for the evaluation of POCU1b on lipolytic activity, the effects of POCU1b on glycerol release, increase in cAMP levels, and PDE activity were investigated. Glycerol release from adipocyte pads was increased by POCU1b in a dose-dependent manner (Figure 2b). We also investigated whether POCU1b affects an adrenoceptor-mediated glycerol output using rat adipocytes. While propranolol, an adrenoceptor antagonist, inhibited isoproterenol-induced glycerol release, it did not inhibit POCU1b-induced glycerol release (Figure 2c). One of the best known mechanisms for lipolysis in the adipocyte is the cAMP-dependent pathway and isoproterenol, one of the stimulators of lipolytic activity via cAMP-dependent signaling [21], was used as a reference in this study. Both isoproterenol and POCU1b significantly induced an increase in cellular cAMP from fat pads (Figure 2d). The inhibitory effect of POCU1b on PDE activity (half-maximal inhibitory concentration (IC_50_): 10.1 μg/mL) was stronger than that of the PDE inhibitor, 3-isobutyl-1-methylxanthin (IBMX) (IC_50_: 667.0 μg/mL) (Figure 2e).

### 3.3. Effects of POCU1b on Body Weight and Food Intake in HFD-Fed Rats

**Figure 3:**
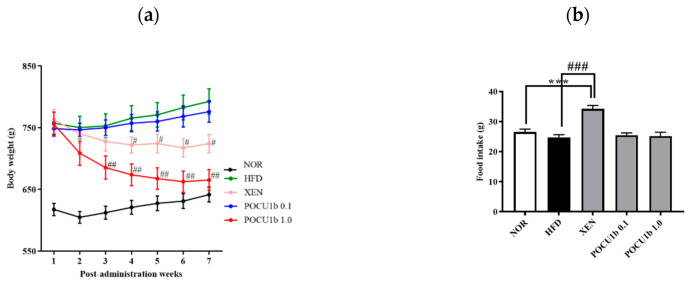
Effects of POCU1b on body weight loss and food intake in high-fat diet (HFD)-fed rats. (**a**) body weight. (**b**) Food intake. After induction of the obese SD rats by feeding with 45% fat for 13 weeks, HFD containing POCU1b or Xenical^®^ (Roche pharma Ltd., Reinach, Swiss) was fed for seven weeks. Each group included 10 rats. Body weight and the total amount of food consumption were measured once a week. NOR: normal diet-fed group; HFD: HFD-fed group; XEN: HFD containing 0.1% Xenical®-fed group; POCU1b 0.1: HFD containing the 0.1% POCU1b-fed group; POCU1b1.0: HFD containing 1% the POCU1b-fed group. *** *p* < 0.001 vs. NOR, ^#^ *p* < 0.05, ^##^ *p* < 0.01, and ^###^ *p* < 0.001 vs. HFD.

As shown in Figure 2, lipolytic actions of POCU1b were confirmed in in vitro, ex vivo, and short-term in vivo experiments. To evaluate the lipolytic effect of POCU1b in a long-term animal study, the obese SD rats were induced by feeding with 45% fat for thirteen weeks, and then POCU1b or Xenical^®^ was fed for seven weeks. From the third week after dietary POCU1b supplementation, POCU1b 1.0 significantly decreased the body weight gain compared to the HFD. The body weight loss effect of POCU1b 1.0 was stronger than that of Xenical® (Figure 3a). Whereas food intake in the XEN group was markedly increased compared to the NOR or HFD groups, POCU1b did not affect food intake (Figure 3b).

### 3.4. Effect of POCU1b on Fat Weight and Adipocyte Size in HFD-Fed Rats

**Figure 4:**
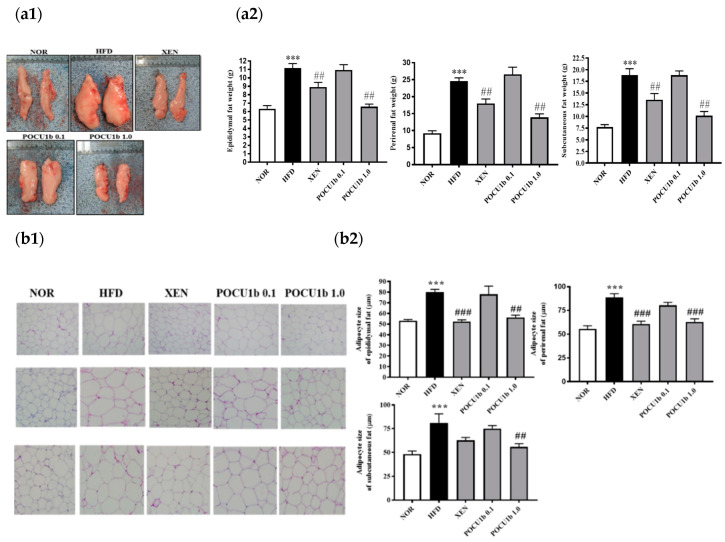
Effects of POCU1b on fat weight and adipocyte size in epididymal, perirenal, and subcutaneous fat tissues in HFD-fed rats. (**a1**) Photographs of epididymal fat for all groups, (**a2**) fat weight, (**b1**) fat tissue morphology, and (**b2**) adipocyte size in epididymal, perirenal, and subcutaneous fat tissues. *** *p* < 0.001 vs. NOR, ^##^ *p* < 0.01, ^###^ *p* < 0.001 vs. HFD.

Compared to the HFD group, in which a dramatic increase in epididymal fat was observed, POCU1b 1.0 markedly suppressed the increase in epididymal fat (Figure 4(a1)). The weight and adipocyte size in epididymal, perirenal, and subcutaneous fat tissues were significantly increased in the HFD group compared to the NOR group, whereas POCU1b 1.0 significantly suppressed these increases compared to the HFD group (Figure 4(a2),b).

### 3.5. Effects of POCU1b on Serum Lipid Levels in HFD-Fed Rats

**Figure 5:**
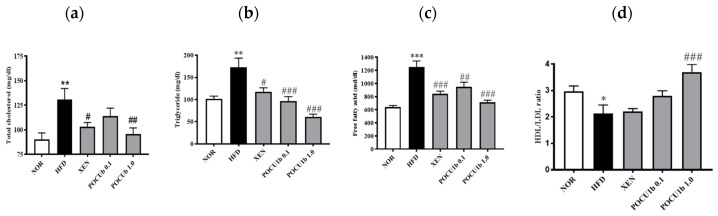
Effects of POCU1b on serum lipid contents in HFD-fed rats. * *p* < 0.05, ** *p* < 0.01, *** *p* < 0.001 vs. NOR, ^#^ *p* < 0.05, ^##^ *p* < 0.01, ^###^ *p* < 0.001 vs. HFD. (**a**–**d**) Serum lipid levels were significantly ameliorated by the treatment with POCU1b in HFD-fed rats.

Serum levels of TC, TG, and free fatty acids were significantly increased in the HFD group compared to the NOR group. POCU1b 1.0 significantly suppressed these increases (Figure 5a−c). The HDL and LDL cholesterol ratio was reduced in the HFD group. However, it was significantly increased in the POCU1b 1.0 compared to the HFD group (Figure 5d).

### 3.6. Effects of POCU1b on NAFL and IR in HFD-Fed Rats

**Figure 6:**
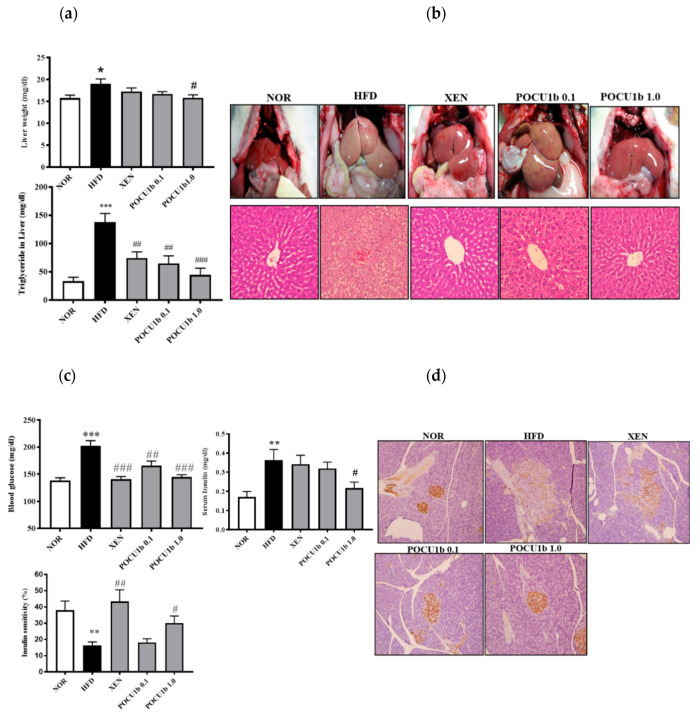
Effects of POCU1b on hepatic lipid accumulation and insulin resistance in HFD-fed rats. (**a**) Liver weight and liver TG content. (**b**) Gross macroscopic evaluation of the liver and photomicrographs of H&E-stained liver tissues. (**c**) Blood glucose, insulin levels, and insulin sensitivity. (**d**) Insulin secretion in the pancreas. * *p* < 0.05, ** *p* < 0.01, *** *p* < 0.001 vs. NOR, ^#^ *p* < 0.05, ^##^ *p* < 0.01, ^###^ *p* < 0.001 vs HFD. (**b,d**) Original magnification 200×.

Liver weight and liver TG levels were increased in the HFD group compared to the NOR group, whereas POCU1b 1.0 significantly inhibited these increases (Figure 6a). In the NOR, XEN, and POCU1b 1.0 groups, liver tissues were red, whereas the livers in the HFD group were extremely pale (Figure 6b upper panel). H&E stained liver tissues in the NOR, XEN, and POCU1b 1.0 groups showed normal polyhedral hepatocytes and many sinusoids. In contrast, the livers in the HFD group showed numerous fat vacuoles. POCU1b 1.0 markedly attenuated the histopathological characteristics of NAFL observed in the HFD group (Figure 6b, lower panel). Fasting blood glucose and serum insulin levels in the HFD group were increased compared to those in the NOR group. They were significantly reduced in the POCU1b 1.0 group compared to the HFD group (Figure 6c upper panel). Although insulin sensitivity was reduced in the HFD group, it was significantly ameliorated by POCU1b 1.0 treatment (Figure 6c, lower panel). Immunohistochemical staining of the pancreatic tissues of the HFD group showed weak insulin immunoreactivity in the islets of Langerhans. The POCU1b 1.0 group showed strong insulin antigen positivity in the majority of the islets of Langerhans (Figure 6d).

### 3.7. Effects of POCU1b on Adipokines in the Serum and/or Liver of HFD-Fed Rats

**Figure 7:**
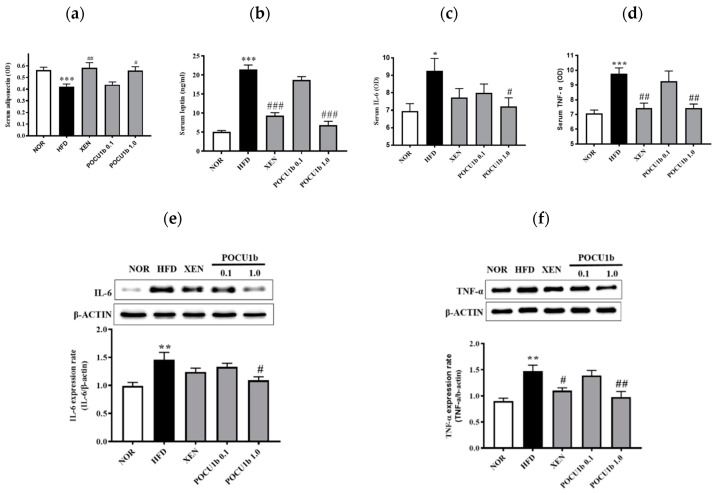
Effects of POCU1b on levels of adiponectin, leptin, IL-6, and TNF-α in the serum and/or liver in HFD-fed rats. (**a**) Serum adiponectin and (**b**) leptin levels. (**c**) IL-6 level in the serum and (**e**) expression in the liver. (**d**) TNF-α level in the serum and (**f**) expression in the liver. Serum adiponectin, leptin, insulin, TNF-α, and IL-6 levels were quantified using commercial ELISA kits. Protein expression was measured by Western blot analysis. * *p* < 0.05, ** *p* < 0.01, *** *p* < 0.001 vs. NOR, ^#^ *p* < 0.05, ^##^ *p* < 0.01, ^###^ *p* < 0.001 vs. HFD.

Serum adiponectin in the HFD group was significantly lower than that in the NOR group. Serum leptin levels were higher in the HFD group than in the NOR group. However, they were significantly restored in the POCU1b 1.0 group to the normal ranges (Figure 7a,b). Levels of IL-6 (Figure 7c,e) and TNF-α (Figure 7d,f) in the serum and liver were significantly increased in the HFD group, whereas POCU1b 1.0 restored them to the normal ranges.

### 3.8. Effects of POCU1b on p-AMPK, AMPK, p-ACC, SOCS-3 Protein, and Fatty Acid Synthase (FAS) mRNA Expressions, and CPT-1 and NF-κB DNA-Binding Activities in HFD-Fed Rat Livers

**Figure 8:**
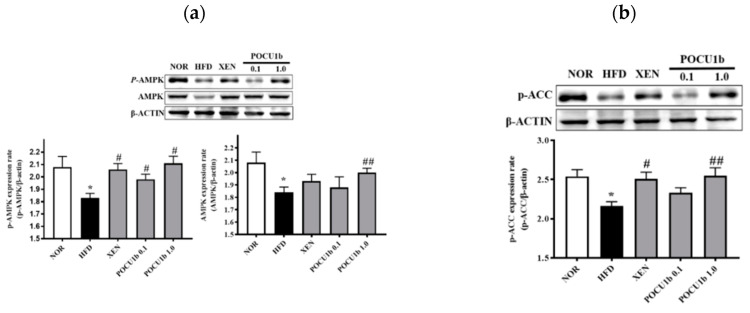

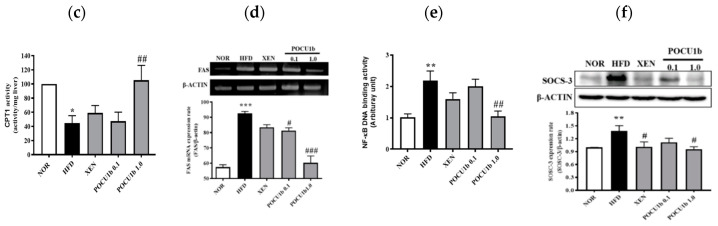
Effects of POCU1b on adenosine monophosphate-activated protein kinase (AMPK), phosphorylated (p)-AMPK, p-acetyl-CoA carboxylase (ACC), suppressor of cytokine signaling (SOCS)-3 protein and fatty acid synthase (FAS) mRNA expression, carnitine palmitoyl tranferase (CPT)-1, and NF-κB DNA-binding activities in HFD-fed rat livers. (**a**) p-AMPK and AMPK levels, (**b**) p-ACC level, (**c**) CPT-1 activity, (**d**) FAS expression, (**e**) NF-κB DNA-binding activity, and (**f**) SOCS-3 protein expression in the livers of rats of all treatment groups. Protein expression was measured by Western blot analysis. Relative mRNA expression level of FAS was measured by RT-PCR. * *p* < 0.05, ** *p* < 0.01, *** *p* < 0.001 vs. NOR, ^#^ *p* < 0.05, ^##^ *p* < 0.01, ^###^ *p* < 0.001 vs HFD.

Protein levels of *p*-AMPK, AMPK, *p*-ACC, and CPT-1 activity were significantly decreased in the HFD group compared to the NOR group. In the POCU1b 1.0 group, these values were restored to the normal ranges (Figure 8a−c). FAS mRNA expression was significantly increased in the HFD group compared to the NOR group; however, POCU1b 1.0 suppressed FAS mRNA expression to the near normal range (Figure 8d). NF-κB DNA-binding activity in the HFD group was increased twofold compared with the NOR group. SOCS-3 expression was also significantly increased in the HFD group compared to the NOR group; however, both were restored to near normal ranges by treatment with POCU1b 1.0 (Figure 8e,f).

## 4. Discussion

In the present study, we investigated the effect and action mechanisms of POCU1b on obesity, obesity-induced non-alcoholic fatty liver (NAFL), and insulin resistance (IR) by using rats infused with lipid emulsion, an HFD-induced obese rats model, and an enzymatic assay. POCU1b effectively decreased the serum TG levels in the rats from 2 h after oral administration of a lipid emulsion (Figure 2a), which means that POCU1b may prevent the uptake of dietary fat from the intestine via the inhibition of pancreatic lipase activity. Stimulation of the β-adrenoceptors in adipocytes leads to increased lipolysis through the production of cAMP, which is followed by activation of protein kinase A (PKA) and hormone-sensitive lipase (HSL). cAMP is an important second messenger in the signaling pathways that mobilize fat stores. PKA proceeds to promote lipolysis by phosphorylation of downstream targets, such as HSL, perilipins, and adipocyte differentiation-related protein (ADRP) [22]. A PDE inhibitor can elevate the intracellular level of cAMP, and the subsequent increase in cAMP stimulates HSL [11]. PDE inhibition also may serve as a therapeutic approach for obesity [23]. POCU1b significantly increased glycerol release and cAMP levels, and showed 60-fold stronger PDE inhibitory activity than IBMX, an inhibitor of cAMP-degrading PDE enzymes (Figure 2b−e). The binding between isoproterenol and adrenoceptor is blocked by propranolol, an adrenoceptor antagonist. Although propranolol inhibited isoproterenol-induced glycerol release, it did not affect POCU1b-induced glycerol release (Figure 2c). POCU1b was confirmed not to be an adrenoceptor agonist, and also did not reduce appetite in HFD-fed rats (Figure 3b). We previously reported that POCU1b inhibits adipocyte differentiation by decreasing lipid droplet formation and expression levels of perilipin and ADRP in 3T3-L1 preadipocytes [17]. It can be extrapolated that POCU1b may act as an anti-obesity agent by inhibiting pancreatic lipase and mediating cAMP-dependent PDE inhibition, leading to reduced perilipin and ADRP levels.

A long-term animal study of POCU1b obviously showed the significant decrease in body weight, and weight and adipocyte size in epididymal, perirenal, and subcutaneous fat tissue (Figure 3 and Figure 4). Obesity-related dyslipidemia is primarily characterized by increased plasma levels of free fatty acids and TG, and a decreased HDL/LDL cholesterol ratio [24]. The hypolipidemic effect of POCU1b 1.0 was confirmed by its suppressive effect on circulating TC, TG, and free fatty acids, and an increased HDL/LDL cholesterol ratio (Figure 5). Increased TG accumulation in the liver preludes the development of advanced non-alcoholic fatty liver disease (NAFLD) [25]. Treatment with POCU1b 1.0 restored the liver weight and liver TG levels to near the normal range, and ameliorated the pathological phenomena, such as pale appearance of livers with hepatocyte vacuoles due to excess fat accumulation (Figure 6a,b).

Adipokines, such as adiponectin, leptin, TNF-α, and IL-6, are thought to influence multiple processes, including glucose and fatty acid metabolism, insulin sensitivity, and adipocyte differentiation, because of their local or peripheral actions in the organism [26]. Alterations in the circulating adipokine level are involved in TG accumulation, which causes liver damage [24]. Adiponectin levels are lower in subjects with NAFLD or IR than in healthy controls [27,28,29]. Overexpression of adiponectin in mice improves dyslipidemia, insulin sensitivity, and glucose tolerance [30,31]. Plasma adiponectin levels were negatively associated with TG and positively associated with HDL cholesterol [32]. Leptin normally inhibits appetite and stimulates energy expenditure. In obese subjects, circulating leptin levels are increased due to the existence of leptin resistance and in response to pro-inflammatory cytokines, such as TNF-α and IL-1, and endotoxin [33]. TNF-α and/or IL-6 are associated with hyper-TG, plasma glucose, or IR [34]. Subjects with hyper-TG have a higher production capacity for IL-6 and TNF-α [35,36,37,38]. POCU1b 1.0 significantly ameliorated the levels of circulating adiponectin and leptin, and serum and liver levels of IL-6 and TNF-α compared to the HFD (Figure 7).

AMPK plays a key role in the regulation of energy homoeostasis and stimulates fatty acid oxidation through phosphorylation of ACC, thereby decreasing malonyl-CoA levels, which activates CPT-1 and increases fatty acid entry into mitochondria [24]. HFD feeding impairs the activity and expression of AMPK and subsequently reduces the p-ACC protein contents [39]. During lipogenesis, FAS is expressed mostly in the liver and adipose tissue with a high rate of fatty acid synthesis [40]. Adiponectin stimulates fatty acid oxidation and glucose utilization via AMPK activation in the liver and skeletal muscle [41]. In 3T3-L1 cells, POCU1b was confirmed to increase the level of p-AMPK. Its effect was stronger than that of hydroxycitric acid, an anti-obesity agent and the active ingredient of *Garcinia cambogia* [17]. The HPLC chromatogram and quantitative analysis of the marker compound in POCU1b revealed resveratrol and emodin derivatives as the major compounds (Figure 1, Table 1). Resveratrol reduced hepatic accumulation of lipids and improved lipid and glycemic metabolism via signaling pathways of AMPK, sirtin 1, and NF-κB [42]. Emodin significantly reduced the lipid deposition in zebrafish livers by regulation of the AMPK signaling pathway, leading to attenuate NAFLD [43]. POCU1b 1.0 increased the adiponectin level, AMPK, p-AMPK, and p-ACC protein levels, as well as CPT-1 activity, and decreased FAS mRNA expression in the liver (Figure 7a and Figure 8a−d), preventing the progression of NAFL (Figure 6a−d).

Insulin initiates its action by binding to its membrane receptor, leading to tyrosine phosphorylation and activation of the insulin receptor. Adiponectin binding to its receptor induces AMPK activation. Activation of the AMPK/tuberous sclerosis complex 1/2 signaling pathway reduces mammalian target of rapamycin/p70 S6-mediated serine phosphorylation of insulin receptor substrate proteins, which results in the enhancement of insulin receptor substrate tyrosine phosphorylation and insulin signaling to activate Akt, leading to glucose uptake [44]. High levels of circulating free fatty acids can also cause peripheral IR in both animals and humans [45], and the amount of fat in the liver is directly related to the degree of IR [46]. Members of the SOCS family are associated with insulin resistance, and their ectopic expression inhibits insulin resistance signaling. Several SOCS proteins are induced by IL-6 and TNF-α in human or rat liver macrophages [47]. Thus, IL-6- or TNF-α-dependent insulin resistance is mediated, at least in part, by the induction of SOCS proteins in insulin target cells [48]. POCU1b 1.0 significantly increased AMPK and p-AMPK levels, and suppressed NF-κB DNA-binding activity and SOCS-3 expression in the liver (Figure 8a,e,f), thereby preventing the progression of IR (Figure 6c,d).

As already published [49,50], the effects of XEN on body weight, TG content in the liver, insulin resistance, and hypolipidemia were confirmed in the present study. Furthermore, XEN supplementation also induced an emergent adverse reaction, like oily spotting, in this study and as previously reported [8,9], which was not observed in POCU1b-treated groups. When compared with the Xenical-treated group, the action of POCU1b on weight loss was confirmed to be more effective than that of Xenical. Furthermore, POCU1b did not show side effects, such as soft stools, flatulence with oily spotting, or loss of appetite.

In conclusion, when combined with our previous studies, we demonstrated that POCU1b inhibited body weight gain and NAFL through inhibition of pancreatic lipase and cAMP-dependent PDE activity, activation of AMPK, ACC, and CPT-1, and the inhibition of FAS mRNA expression. Simultaneously, POCU1b inhibited the development of obesity-induced IR via AMPK activation, and the inhibition of NF-κB DNA-binding activity and SOCS-3 overexpression. Based on these results, we suggest that POCU1b possesses therapeutic and/or preventive potential for obesity, NAFL, and IR, without critical discomfort, such as oily spotting and loss of appetite.

## 5. Patents

The patents related to this study were registered in Korea (no. 10+1764786), Germany, England, Swiss (no. 2520307), China (no. ZL200980162324), Israel (no. 218855), EU (no. 2520307) and the USA (no. 954725).

## Figures and Tables

**Table 1:** Contents of marker compounds in POCU1b.

Compound	Content (Mean ± SD, *n* = 3)
	mg/g
Resveratrol-3-*O*-β-d-glucopyranoside	43.68 ± 2.21
Resveratrol	1.37 ± 0.07
Emodin-1-*O*-β-d-glucopyranoside	4.71 ± 0.28
Torachrysone-8-*O*-β-d-glucopyranoside	5.99 ± 0.28
Emodin-8-*O*-β-d-glucopyranoside	62.91 ± 3.22
Physcion-8-*O-*β-d-glucopyranoside	18.82 ± 0.96
Emodin	7.20 ± 0.33
